# Letters on Syphilis, No. 1.

**Published:** 1851-12

**Authors:** 


*Letters on Syphilis, No.* 1. By M. P. RICORD, Chirurgien de l'Hopital du Midi, Paris. Translated by W. P. LATTIMORE, M. D.

To Dr. Amedee Latour, Editor of the "Union Medicale."

*My dear Confrere and Friend:*— The new doctrine on Syphilis experiences the fate of every scientific discovery. For nearly twenty years, by my teaching and by my writings, I have sought to make it penetrate the minds of my contemporaries. I see, however, that it is not equally understood by all; certain adversaries still make objections to it — objections which I have a hundred times refuted; — and, what is more curious, certain others seize upon objections raised by myself, and imagine, a little naively, perhaps, to vanquish me with arguments introduced into this discussion by myself.

At this I am neither astonished nor indignant. On the contrary, I find in it a new inducement to continue my work; and far from complaining of my adversaries, I rather thank them for holding my zeal on the alert, thus preventing it from flagging.

Thus, I am about to demand your permission to make an exposition of the true doctrines of the "Hopital du Midi," in your widely circulated Journal. Allow me to say, that it is less an individual response, than a general exposition, which I intend to make. As I proceed, I shall encounter objections, and will endeavor to reply to them; I will also bear in mind, as far as I ought, a recent publication, due to the pen of one of your skillful co-laborers, who, in order to find *followers*, need not have gone modestly *to the country* to seek them.

I will present you, my dear friend, a preliminary reflection inspired by the publication to which I have just alluded; — because an observer may not be permitted to see all of the facts of a particular part of pathology, and to arrange a general system, we must not hence conclude that this observer has accomplished nothing, has seen nothing, has established nothing; that his labors and his researches ought to be regarded as naught, and a clean sweep made of his teaching. This method of philosophizing in medicine, perhaps a little too common at this day, is convenient and expeditious, but is neither true nor just. In Syphilography, particularly, this process would lead to deplorable errors. A serious study of the history of our art demands more moderation of language, more justice of appreciation. For my part, I am pleased to recognize and to proclaim, that instead of all Syphilographic literature being worthy of contempt, there is to be found in it, by those who know how to see them, interesting and curious observations, good precepts, and, even sometimes doctrinal absurdities which some find it good to exhume, while they discredit the source. Of a surety, the long discussions on Mercury, Guiac, Sarsaparilla, etc., are not wholly devoid of utility; the history of Blennorrhagia may be cleared up by the observations of those who have preceded us. Undoubtedly, the spirit of speculation and charlatanism have left too frequent traces of their passage, but you will often find in them also, the imprint of judicious spirits, of a veritable scientific tendency, and of laudable efforts to arrive at a systemization and a doctrine. These labors, besides. had they no other interest than that of reflecting the ideas and opinions of past times, would not merit the contempt which has been cast upon them unjustly.

I would make the same profession of faith in regard to observers of modern times. Criticism, I know by experience, finds frequent occasions to exercise itself upon their works. But, must we hence consider them unimportant? Far from me be this injurious thought. On the contrary, I hold in great esteem the writings of Bell, of John Hunter, of Swediaur; the time has come to render full justice to the two Culleriers, to M. Lagneau, particularly, whose reputation was justly popular; to all those intelligent and laborious workmen of our science who, by conscientious studies, have, with much pains, opened the way in which we can advance more freely.

Should I be unjust towards my contemporaries? God forbid, dear friend. Whatever may be our differences, it is spontaneously and with pleasure, that I render the most sincere homage to the works of MM. Baumes, Gibert, Cazenave, Cullerier, (the nephew,) Bottex, Ratier, Pache, Diday, Payan, Venot, in France; abroad, Wallace, Carmichael, Babington, and my pupils Acton and Meric, in England; Thiry, Herion, in Belgium; to the remarkable publications of laborious Germany and of ingenious Italy.

I do not then experience, either towards the past, or towards the present, any sentiment of injustice or of disdain. You will excuse me for making this declaration very explicitly before entering upon the subject. I think it proper to say, that I partake in no manner of the opinion of those exacting and difficult critics, according to whom both ancient and modern Syphilography is only a medley unworthy of attention. I believe, on the contrary, that this branch of pathology is as fertile as any other in useful works, and in precious researches.

However, the works of the ancients and moderns have not been able to preserve this part of our science from the general revolutions impressed upon medicine by the physiological doctrine. The school of Broussais, in blotting out the past, had put everything in doubt. Was there a syphilitic virus? Did Syphilis exist? You know how physiologism resolved these questions. The most extreme confusion reigned in science, and was transferred into the publications of the time. Doubt was every where, certainty no where.

It was at this epoch that, Surgeon by "concours" to the Central Bureau of the Hospitals, it was my lot to enter the Hopital du Midi. I there met an honest and loyal man, a serious and honest practitioner. M Cullerier, who, having abandoned the family traditions, so to speak, had taken upon himself to doubt his own observation, and appeared no longer to believe what he had seen.

Every where doubt had replaced belief; people doubted the cause of Syphilis, doubted its effects, and, as a consequence, they doubted its therapeutics.

And mark! that which was called the new doctrine, was presented, surrounded by a great scientific apparatus. M. Richoud des Brus wrote an enormous book completely filled with facts; M. Desruelles supported the new idea by statistics which passed for exact; all strove to combat the specialty of the disease and the specific nature of the remedy.

History was largely laid under contribution by one of the most learned writers of our century, M. Jourdan, who, in one of the most remarkable works of our epoch, was pleased to take the observers, one by one, and to put them in contradiction with themselves; an easy triumph, if the critic, in an austere and impartial analysis, does not know how to establish a marked difference between the ideas proper to the author, those which result from his researches and his observation, and those which he draws from the scientific media of his time. The first are useful materials which it is necessary to preserve; the others constitute the prejudices of the epoch, and have only an historical value. Jourdan did not take this precaution; it sufficed him, in order to combat the specific nature of the disease, to indicate the confusion of opinion among our predecessors, and he did it with a luxury of erudition which would have been an ornament to a more healthy criticism.

Such, then was the state of opinion and of science, when I entered the "Hopital du Midi." It was necessary to rebuild, according to some, a ruined edifice; it was at least necessary, according to others, to consolidate it.

That which was most of all, necessary, was to take up the study of the cause of Syphilis.

Had it a special cause, a virus? Or, were venereal accidents the result of a common cause?

For this research and this study, *two methods* of observation offered themselves to me.

The *first* was the pure and simple observation of the phenomena, the observation which our predecessors had practiced, and which had conducted them to opinions so divergent; the observation similar to that of Devergie, and altogether analogous to the facts already reported by Vigaroux, by Bligny, etc.; that observation, for example, relative to three officers, who had connection with the same young girl, affected with a discharge, and were all three infected; one, with an urethritis, the second, with a chancre, and the third, with warts. It is true that Devergie deprived the history of information.

on one small point, that of the precise state of the young girl, whom he did not examine with the *speculum.*

Evidently, this mode of investigation was worn out, and could conduct only to vagueness, or to a confusion in the results.

The *second method* was more satisfactory to my reason; it was, besides, more in affinity with the demands of modern science; it seemed to me to open a sure way to the study, and of necessity to lead to incontestible results. I speak of EXPERIMENT.

I laid down for myself the following *conditions:*

To derive the cause of Syphilis from a known source;

To place it upon a region of the body open to observation;

To note its effects.

You see that experiment alone could fulfil these conditions.

But experiment had already been interrogated, and by it people had arrived at contradictory conclusions. When John Hunter said yes, Caron, Bru, Jourdan, Devergie, and M. Desruelles said no. Upon what could depend affirmation so opposite, when the same method of investigation had been employed. I did not then know, I have since learned. What my reason then told me was, that a series of rigorous and well-conducted experiments must lead to precise results; and the dissensions of experimenters did not dishearten me.

These researches were difficult and delicate. There was needed convictions, and I dare to say, courage to undertake them; it was necessary to be certain of well appreciating the circumstances under which I was about to act; it was necessary to rely upon antecedent experiments; it was particularly necessary to rely upon purity of intention, and upon the testimony of the conscience.

I did not, in fact, content myself with the great name of Hunter; with the experimenters cited by Bell; with the work of Hernandez, crowned by the academy of Besançon, with the authority of Percy, and with some other great names equally renowned; but I wished to study the question in itself, to place myself in the conditions of a veritable inventor, in fact, to assume upon myself alone all the responsibility of the results.

How was it necessary to proceed to this experimentation?

I could inoculate from a diseased to a healthy individual; I could experiment on the patient himself.

The first method of experimenting, that is, the inoculation from a diseased to a healthy individual, it appeared to me, ought always to be rejected by the physician. I do not believe we have the right to make such experiments. The physician not only ought not to use his natural authority to instigate any one to undergo experiments of this nature, but I farther think, that he ought to resist the desires of individuals, who, seduced by a generous devotion, would voluntarily expose themselves to the chances of an inoculation. I cast no blame on those who have acted differently. I only repeat that for my part, I have been unwilling to proceed thus. There remained to experiment upon the patient himself.

Could this present inconveniences and dangers for the patient?

In the event of its harmlessness, could it lead to conclusive results?

Here is what history, observation, and experience, teach on this subject.

It was generally admitted that a first contagion did not prevent a second one, and the old saying of *pox on pox,* still had full force. At this day we know what to understand by that.

As to the inconveniences and dangers, we see every day that primitive accidents are rarely isolated; that they are multiplied with a great facility, and that strictly, the gravity of the disease is not in relation to the number of accidents.

Then in order to elucidate so grave a question of etiology and of practice, art could, without inconvenience, do what nature habitually does.

A much graver question here presented itself: Were the profound and consecutive accidents to be feared from infection in proportion to the number of the primitive lesions?

Rigorous clinical observation of every time has proved, and proves every day, that constitutional Syphilis is not in proportion to the number of primitive accidents, *existing at the same time, developed at the same epoch.*

An additional accident then adds nothing to the chance of infection, *if we know how to direct the experiment.*

There remained the question of *surface;* to know whether an extensive ulceration exposed more to a general infection than an ulceration of moderate size. Here, again, observation had shown that a more or less extended surface of a primitive ulceration has no influence upon the production of consecutive accidents. A very small chancre exposes to a general infection, just as much as a very extensive chancre; and reciprocally, a large ulceration exposes one no more nor less than a small one.

Finally, there remained the question of the seat of the ulceration, of the place of election for the experimental punctures. It had been said, and by Boerhaave, among others, that venereal accidents contracted elsewhere than on the genital organs, presented a greater degree of gravity; but clinical observation had already proved, and it has since proved to me, that this opinion was erroneous.

I know very well, that on this point a great noise has been made in regard to diseases contracted by physicians and sages femmes, the result of examinations, punctures, etc. There are very good reasons which I do not wish to give here, why these accidents should have made a great noise. I can say, however, without wounding any, that members of the profession to whom such accidents happen, have no motive for concealment, while ordinary syphilitic patients have excellents for saying nothing.

I rested then convinced that the seat of the ulceration could not only have no unfavorable influence upon the production of consecutive accidents, but that it might even diminish or destroy certain troublesome casualties, for example, the production of buboes. Thus, observation had already proved, that primitive chancres of the thigh were almost never followed by adenitis; and, in fact, among my numerous experiments, I have never seen an adenitis supervene upon the punctures of inoculation on the thigh.

Then, my dear friend, from history, from clinical observation of every age, from the experimenters who had preceded me, from the testimony of my conscience, severely interrogated, I arrived at this encouraging conclusion, that in experimenting on the patient himself,

I would in reality give him no more disease;

I would not augment the gravity of the accidents with which he was already affected;

I would not expose him to any greater chances of a consecutive infection.

These first and capital conditions being found, it was necessary to seek for those which should offer to science and to art, every desirable guarantee.—*N. Y. Med. Times.*

